# Association between undercarboxylated osteocalcin, bone mineral density, and metabolic parameters in postmenopausal women

**DOI:** 10.20945/2359-3997000000061

**Published:** 2018-08-01

**Authors:** Leila C. B. Zanatta, Cesar L. Boguszewski, Victoria Z. C. Borba, Carolina A. Moreira

**Affiliations:** 1 Universidade Federal do Paraná Universidade Federal do Paraná Divisão de Endocrinologia (SEMPR) Departamento de Medicina Interna Curitiba PR Brasil Divisão de Endocrinologia (SEMPR), Departamento de Medicina Interna, Universidade Federal do Paraná (UFPR), Curitiba, PR, Brasil; 2 Fundação Pró-Renal Seção de Histomorfometria Óssea Laboratório PRO Curitiba PR Brasil Laboratório PRO, Seção de Histomorfometria Óssea, Fundação Pró-Renal, Curitiba, PR, Brasil

**Keywords:** Undercarboxylated osteocalcin, metabolic syndrome, glucose, energy metabolism, diabetes mellitus, bone mineral density

## Abstract

**Objective:**

Osteocalcin has been associated with several effects on energy and glucose metabolism. However, the physiological role of undercarboxylated osteocalcin (U-osc; the hormonally active isoform of osteocalcin) is still controversial. To correlate the serum levels of U-osc with bone mineral density (BMD) values and metabolic parameters in postmenopausal women.

**Subjects and methods:**

Cross-sectional study including 105 postmenopausal women (age 56.5 ± 6.1 years, body mass index [BMI] 28.2 ± 4.9 kg/m^2^) grouped based on the presence of three or less, four, or five criteria of metabolic syndrome according to the International Diabetes Federation (IDF). The subjects underwent dualenergy x-ray absorptiometry (DXA) for the assessment of body composition and BMD and blood tests for the measurement of U-osc and bone-specific alkaline phosphatase (BSAP) levels.

**Results:**

The mean U-osc level was 3.1 ± 3.4 ng/mL (median 2.3 ng/mL, range 0.0-18.4 ng/mL) and the mean BSAP level was 12.9 ± 4.0 ng/mL (median 12.1 ng/mL, range 73-24.4 ng/mL). There were no associations between U-osc and BSAP levels with serum metabolic parameters. Lower fasting glucose levels were observed in participants with increased values of U-osc/femoral BMD ratio (3.61 ± 4 ng/mL versus 10.2 ± 1.6 ng/mL, *p* = 0.036). When the participants were stratified into tertiles according to the U-osc/ femoral BMD and U-osc/lumbar BMD ratios, lower fasting glucose levels correlated with increased ratios (*p* = 0.029 and *p* = 0.042, respectively).

**Conclusion:**

Based on the ratio of U-osc to BMD, our study demonstrated an association between U-osc and glucose metabolism. However, no association was observed between U-osc and metabolic parameters.The U-osc/BMD ratio is an innovative way to correct the U-osc value for bone mass.

## INTRODUCTION

Some studies over the past decade have shown that bone is associated with important physiologic mechanisms involving the control of energy metabolism and glucose homeostasis, attracting great interest in the understanding of the role of bone as an endocrine organ (1–9).

Osteocalcin is a protein produced by osteoblasts and present in two forms, carboxylated and undercarboxylated (U-osc). The carboxylated form is synthesized and stored in the mineral matrix, while U-osc is produced from the degradation of bone matrix and released into the circulation as a hormonally active isoform (2). U-osc has been shown to stimulate cell proliferation and increase insulin production. In addition, U-osc stimulates adiponectin expression in adipose cells, which in turn increases insulin sensitivity and energy metabolism (10).Insulin has direct participation in the process of decarboxylation of osteocalcin, allowing the formation and release of metabolically active U-osc (11). These actions together reveal a positive feedback mechanism involving pancreatic beta cells, adipose tissue, and bone.

Some epidemiological studies have supported the hypothesis of a positive effect of osteocalcin on glucose and energy metabolism (12–23). However, the association of these metabolic parameters with U-osc, in particular, remains elusive (24–36). Based on these facts, the aim of the present study was to correlate serum levels of U-osc and bone mineral density (BMD) with metabolic parameters in a group of healthy, postmenopausal women.

## SUBJECTS AND METHODS

The recruitment for the study occurred between February 2012 and March 2013. The inclusion criteria comprised female gender, postmenopausal status (amenorrhea for more than 1 year), and age below 70 years. The exclusion criteria included diabetes mellitus; renal, hepatic or cardiac dysfunction; bone disease or secondary osteoporosis; cancer; and use of hypoglycemic agents, glucocorticoids, anticonvulsants or antiresorptive drugs.

**Figure 1:**
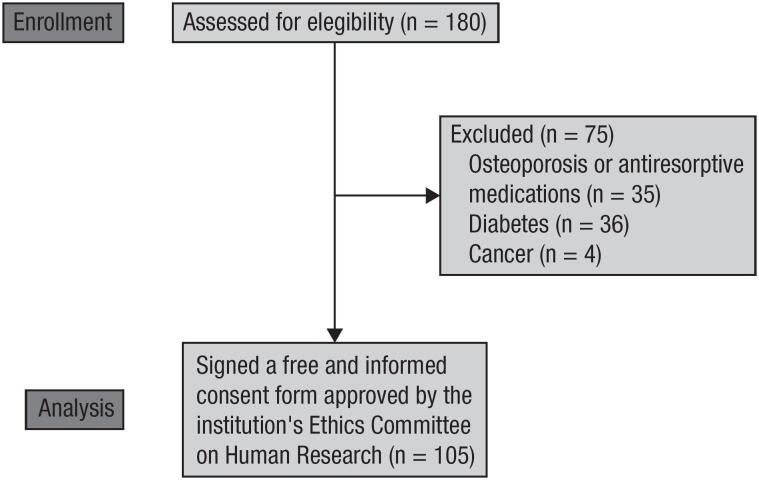
Study Flowchart.

A total of 180 women were evaluated. Of these, 35 were excluded due to a diagnosis of osteoporosis based on densitometric values and use of antiresorptive medications, 36 due to a diagnosis of diabetes, and 4 due to a diagnosis of cancer (Figure 1).

All participants signed a consent form approved by the institution's Ethics Committee on Human Research.

The participants underwent a clinical evaluation and were classified according to clinical parameters, including body mass index (BMI), waist circumference, and blood pressure, which followed the 2005 classification of the International Diabetes Federation (IDF 2005) (37). Values of BMI were considered normal if between 18.5-24.99 kg/m^2^, overweight if 25-29.99 kg/m^2^, class I obese if 30-34.99 kg/m^2^, class II obese if 35-39.99 kg/m^2^, and class III obese if greater than 40 kg/m^2^.

Blood samples were collected between 8:00 and 9:00 am after an 8-hour overnight fast. Levels of U-osc were measured with the Glu-OC EIA Kit (Takara Bio Inc., Otsu, Japan; reference values 0.25-8 ng/mL, intra-assay and interassay variations 4.4% and 5.67%, respectively). For this measurement, blood was collected in ice-cold tubes and immediately centrifuged in a cold centrifuge, and the serum was stored in cryotubes at −80°C. Levels of bone-specific alkaline phosphatase (BSAP, a specific marker of bone formation) were determined with the LIAISON® BAP OSTASE® assay (normal range 15-120 pg/dL, intra-assay variation 4.5%) simultaneously to those of U-osc in order to better evaluate the hormonal action of osteocalcin on bone metabolism.

Glucose was measured by a hexokinase/glucose-6-phosphate dehydrogenase method (normal values < 99 mg/dL) and insulin by chemiluminescence (normal values 2.5-30 μUI/mL). The homeostasis model assessment of insulin resistance (HOMA-IR) was used to measure insulin sensitivity using the following formula: (fasting glucose in mg/dL X fasting insulin in UI/mL) / 405 (38). The methods used to measure the remaining variables and their respective normal values are as follows: C-reactive protein (CRP) - turbidimetry (normal values < 0.5 mg/dL); high-density lipoprotein (HDL)-cholesterol - direct, homogeneous method (low values ≤ 40 mg/dL and high values ≥ 60 mg/dL); triglycerides (TG) - glycerol phosphate oxidase (optimal values < 150 mg/dL, borderline values 150-200 mg/dL, increased values > 200 mg/dL); creatinine - alkaline picrate method (normal values 0.57-1.11 mg/dL); calcium - Arsenazo III (normal values 8.6-10.3 mg/dL); phosphorus - UV phosphomolybdate (normal values 2.3-4.7 mg/ dL); PTH - chemiluminescence (normal values 11.761.1 pg/mL); 25-hydrox yvitamin D3 - CLIA (normal values 30-100 ng/mL); alanine aminotransferase (ALT) and aspartate aminotransferase (AST) - colorimetric method (normal values 0-55 IU/L and 5-34 IU/L, respectively).

All patients were evaluated in regards to the presence of criteria for metabolic syndrome and grouped according to the number of criteria present as (A) three or fewer, (B) four, or (C) five. According to the IDF 2005 recommendations, the participants were deemed as having a normal waist circumference when this measurement was below 80 cm, and normal blood pressure when the values were below 130/85 mmHg. The finding of serum glucose levels below 100 mg/dL, TG below 150 mg/dL, and HDL-cholesterol above 50 mg/dL was also considered normal.

The participants were also evaluated and categorized according to parameters of body composition. Measurements of lumbar spine (L1 to L4) and total femoral BMD, as well as those of body composition (percentage total fat), were performed with a properly calibrated Lunar Prodigy Advance (GE Healthcare, Madison, WI, USA), with a coefficient of variation of 2% and by the same examiner.

The ratios U-osc/lumbar BMD and U-osc/femoral BMD were calculated using absolute U-osc (ng/mL) values divided by the BMD observed in the lumbar and total femoral regions (g/m^2^), respectively.

## Statistical analysis

Serum U-osc and BSAP levels, as well as the ratios U-osc/ femoral BMD and U-osc/lumbar BMD were compared with the following metabolic parameters: BMI, waist circumference, systolic and diastolic blood pressure, and total fat percentage assessed by dual-energy x-ray absorptiometry (DXA; total fat), TG, HDL-cholesterol, fasting glucose, insulin, HOMA-IR, and CRP. Levels of PTH, 25-hydroxyvitamin D3, calcium, inorganic phosphorus, albumin, AST, ALT, and creatinine were measured to exclude bone, kidney, and liver disease.

The data are described as mean and standard deviation values unless otherwise stated. The MannWhitney and Kruskal-Wallis tests were used for comparisons between groups. The chi-square test was used to estimate the association between U-osc categorized into tertiles and parameters of metabolic syndrome. Correlations between U-osc and the ratios U-osc/lumbar BMD and U-osc/femoral BMD with values of metabolic variables and BMD were estimated using Spearman's rank correlation coefficient. A *p* value below 0.05 was considered significant.

## RESULTS

The clinical characteristics and results of biochemical tests of the study participants are summarized in Table 1. A total of 76 women (72.4%) presented three or fewer criteria for metabolic syndrome, while 18 (17.1%) had four criteria, and 11 (10.5%) had all five criteria for the syndrome. Osteopenia was observed in 57 (54%) participants, while osteoporosis was observed in 21 (20%) of them. When the participants were categorized according to BMI values, 26 (25%) had normal weight, 44 (42%) had overweight, 22 (21%) had class I obesity, and 13 (12%) had class II or III obesity. There were no significant correlations observed between U-osc levels and metabolic parameters, percent total fat, or CRP levels.

**Table 1:** Clinical characteristics and results of biochemical tests of 105 postmenopausal women included in the study

Variable	Mean	Median	SD	Range
Age (years)	56.5	56.0	6.1	43.0 – 70.0
BMI (kg/m^2^)	28.2	27.4	4.9	19.7 – 41.1
Waist circumference (cm)	91.7	90.0	11.3	66.0 – 122.0
Total body fat (%)	41.2	41.7	6.6	23.7 – 54.9
SBP (mmHg)	127.4	120	11.26	90 – 190
DPB (mmHg)	78.8	80	19.57	60 – 110
Triglycerides (mg/dL)	126.7	107.5	58.0	43.0 – 319.0
HDL-cholesterol (mg/dL)	47.8	46.0	12.3	29.0 – 100.0
Blood glucose (mg/dL)	92.7	91.0	10.6	65.0 – 126.0
Insulin (μUI/mL)	10.5	9.1	5.9	2.9 – 33.0
HOMA-IR	2.5	2.0	1.6	0.6 – 9.0
CRP (mg/dL)	0.53	0.33	0.71	0.33 – 6.73
25-hydroxyvitamin D3 (ng/mL)	21.1	20.7	7.0	7.5 – 55.0
U-Osc (ng/mL)	3.1	2.3	3.4	0.0 – 18.4
BSAP (μg/dL)	12.9	12.1	4.0	7.3 – 24.4

SD: standard deviation; BMI: body mass index; SBP: systolic blood pressure; DBP: diastolic blood pressure; HDL-cholesterol: high-density lipoprotein cholesterol; HOMA-IR: homeostasis model assessment of insulin resistance; CRP: C-reactive protein; U-Osc: undercarboxylated osteocalcin; BDAP: bone-specific alkaline phosphatase.

**Figure 2:**
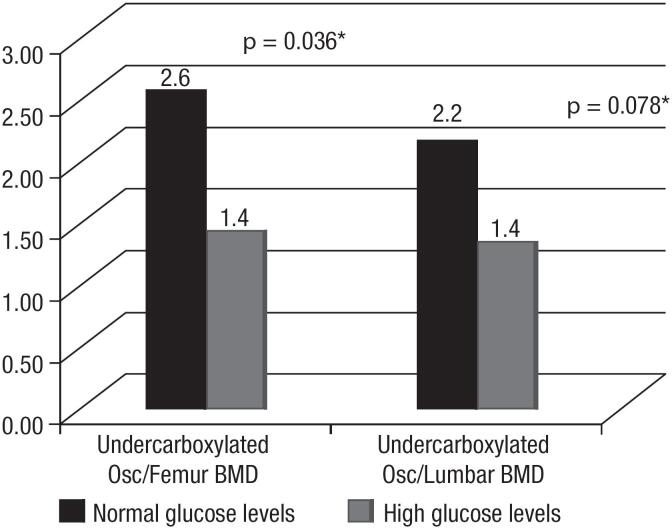
Relationship between the median values of the ratio undercarboxylated osteocalcin (U-osc)/lumbar body mineral density (BMD) and U-osc/femoral BMD with glucose levels in postmenopausal women. Normal glucose values = below 99 mg/dL; high glucose values = equal to or above 100 mg/dL. * Mann-Whitney test; *p* < 0.05.

The median values of the ratio U-osc/femoral BMD were significantly different in participants with normal (< 99 mg/dL) compared with those with increased (> 100 mg/dL) blood glucose levels (2.6 mg/dL and 1.4 mg/dL, respectively, *p* = 0.036), as demonstrated in Figure 2.

**Figure 3:**
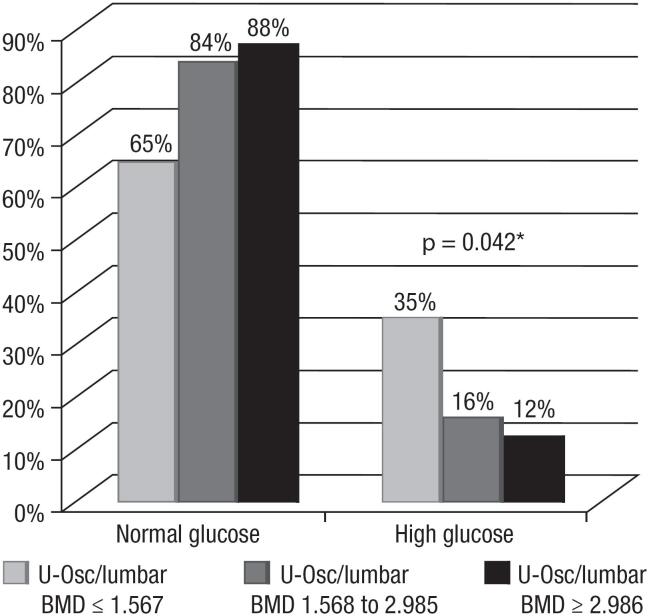
Percentage of women with ratios of undercarboxylated osteocalcin (U-osc)/lumbar body mineral density (BMD), categorized into tertiles according to blood glucose levels. * Chi-square test; *p* < 0.05.

**Figure 4:**
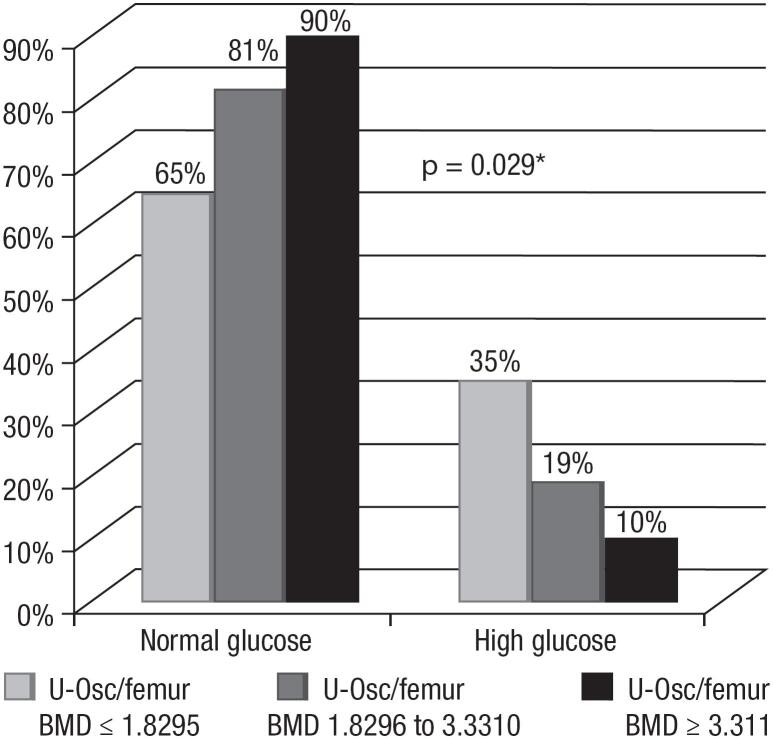
Percentage of women with ratios of undercarboxylated osteocalcin (U-osc)/femoral body mineral density (BMD) categorized into tertiles according to blood glucose levels. * Chi-square test; *p* < 0.05.

When the ratios U-osc/lumbar BMD and U-osc/femoral BMD were categorized into tertiles, participants in the upper tertiles of both ratios had lower fasting glucose levels. Among participants with lumbar ratio values above 2.985 (ng.mL)/(g/m^2^), only 12% presented increased glucose levels (*p* = 0.042) (Figure 3), while for those with femoral ratio values equal to or greater than 3.31 (ng/mL)/(g/m^2^), only 10% had increased glucose levels (*p* = 0.029) (Figure 4).

No correlation was observed between U-osc and femoral or lumbar BMD values (r = 0.122, *p* = 0.218 and r = 0.058, *p* = 0.560, respectively). Similarly, the correlation between fasting glucose levels with femoral and lumbar BMD was not significant (r = 0131, *p* = 0.186 and r = 0.033, *p* = 0.737, respectively).

Levels of BSAP were not significantly associated with BMD values or metabolic parameters.

## DISCUSSION

The results of this cross-sectional study show an inverse association between the ratio U-osc/femoral BMD with blood glucose levels, which was demonstrated by greater ratio values in the subgroup of patients with normal glucose levels. Similarly, when the ratios (lumbar and femoral) were stratified into tertiles, there was an inverse association of both ratios with blood glucose levels. The U-osc/BMD ratio is an innovative way to correct t U-osc values according to the patient's bone mass and was applied in this study in a similar way that leptin/fatty tissue ratio was applied in a study by Paz-Filho and cols. (39). Since osteocalcin is a specific bone protein that is increased in postmenopausal, resorptive conditions, we believe that the correction of osteocalcin values by the patient's BMD yields a more adjusted parameter of the involvement of bone on energy and glucose metabolism.

Similar results were not observed for BSAP, a marker exclusively related to bone and without metabolic effects. Additionally, no correlation of BMD values and U-osc or glucose alone was observed, which allows us to infer that these independent variables had no influence on osteocalcin values.

Similarly to our findings, Shea and cols. and Mori and cols. found no association between U-osc and indices of insulin resistance (30,31). Additionally, Kanazawa and cols. evaluated women with diabetes and found no association between U-osc and blood glucose parameters (25).

Another group of researchers found no association between metabolic syndrome and U-osc (32). Schafer and cols. reported that anabolic treatment for osteoporosis with PTH(1-84) increased U-osc levels, but showed no correlation with increased leptin levels (33).

It is important to point out that the studies focused on U-osc differ from those assessing total osteocalcin, which has been associated with metabolic and atherosclerotic parameters in diabetic and nondiabetic populations of both genders (12–23). An important limitation of studies with U-osc is a lack of homogeneity and specificity of commercially available assays to detect this specific osteocalcin isoform (34–36).

In contrast to our findings, Hwang and cols. found that tertiles of U-osc values had a significant inverse correlation with blood glucose levels and insulin sensitivity (24). Other authors have also shown an inverse association between U-osc levels and fasting glucose, HbA1c, total body, and visceral fat in 180 diabetic men (25). Diaz-Lopez and cols. found that lower levels of total osteocalcin and U-osc were independently associated with a higher risk of diabetes mellitus in individuals with increased cardiovascular risk (26). Pollock and cols. evaluated 140 children with and without prediabetes and observed that children with prediabetes had lower total osteocalcin and U-osc levels, along with worst indices of insulin secretion (27,28). Yeap and cols. showed that increased U-osc level was both a marker of bone turnover and an independent predictor of reduced diabetes risk. Additionally, the increased rate of bone remodeling in senescence could be associated with a reduced risk of diabetes mellitus (29).

The limitations of the present study include the small number of women with increased blood glucose levels (only 24 of the 105 participants), class II and III obesity (only 13 participants), and of those with all criteria for metabolic syndrome (11 participants). Physical activity (resistive or aerobic), which could influence U-osc levels and BMD values (42,43), was not considered in the statistical models. The oral glucose tolerance test and the hyperinsulinemic-euglycemic clamp, which are superior tests in evaluating insulin resistance compared with the HOMA-IR index, were not performed. The cross-sectional evaluation of this study does not allow us to infer a causal relationship between U-osc levels and metabolic parameters.

In conclusion, the results of this study show that the ratios U-osc/lumbar BMD and U-osc/femoral BMD had a significant inverse association with blood glucose levels in a group of nondiabetic, postmenopausal women. This finding reinforces the link between glucose and bone metabolism.

## References

[1] Clemens TL, Karsenty G. The osteoblast: an insulin target cell controlling glucose homeostasis. J Bone Miner Res. 2011;26(4): 677-80. 10.1002/jbmr.321 21433069

[2] Lee NK, Sowa H, Hinoi E, Ferron M, Ahn JD, Confavreux C, et al. Endocrine regulation of energy metabolism by the skeleton. Cell. 2007;130(3):456-69. 10.1016/j.cell.2007.05.047 PMC2013746 17693256

[3] Ducy P. The role of osteocalcin in the endocrine cross-talk between bone remodelling and energy metabolism. Diabetologia. 2011;54(6):1291-7 10.1007/s00125-011-2155-z 21503740

[4] Zanatta LC, Boguszewski CL, Borba VZ, Kulak CA. Osteocalcin, energy and glucose metabolism. Arq Bras Endocrinol Metabol. 2014l;58(5):444-51. 10.1590/0004-2730000003333 25166034

[5] Crier PE. Glicemic and hipoglicemic homeostasis. In: Kronenberg HM, Melmed S, Polonsky KS, Larsen PR. Williams Textbook of Endocrinology, 11th ed. 2009. p. 1190-6.

[6] Galic S, Oakhill JS, Steinberg GR. Adipose tissue as an endocrine organ. Mol Cell Endocrinol. 2010;316(2):129-39. 10.1016/j.mce.2009.08.018 19723556

[7] Ducy, P Amling M,Takeda S, Priemel M, Schilling AF, Beil FT, et al. Leptin inhibits bone formation through a hypothalamic relay: a central control of bone mass. Cell. 2000;100(2):197-207. 10.1016/s0092-8674(00)81558-5 10660043

[8] Cohen B, Novick D, Rubinstein M. Modulation of insulin activities by leptin. Science. 1996;274(5290):1185-8. 10.1126/science.274.5290.1185 8895466

[9] Ducy P, Desbois C, Boyce B, Pinero G, Story B, Dunstan C, et al. Increased bone formation in osteocalcin-deficient mice. Nature. 1996;382(6590):448-52. 10.1038/382448a0 8684484

[10] Karsenty G, Oury F. The central regulation of bone mass, the first link between bone remodeling and energy metabolism. J Clin Endocrinol Metab. 2010;95(11):4795-801. 10.1210/jc.2010-1030 21051575

[11] Lee NK, Karsenty G. Reciprocal regulation of bone and energy metabolism. Trends Endocrinol Metab. 2008;19(5):161-6. 10.1016/j.tem.2008.02.006 18407515

[12] Im JA, Yu BP, Jeon JY, Kim SH. Relationship between osteocalcin and glucose metabolism in postmenopausal women. Clin Chim Acta. 2008;396(1-2):66-9. 10.1016/j.cca.2008.07.001 18657532

[13] Kindblom JM, Ohlsson C, Ljunggren O, Karlsson MK, Tivesten A, Smith U. Plasma osteocalcin is inversely related to fat mass and plasma glucose in elderly Swedish men. J Bone Miner Res. 2009;24(5):785-91. 10.1359/jbmr.081234 19063687

[14] Pittas AG, Harris SS, Eliades M, Stark P, Dawson-Hughes B. Association between serum osteocalcin and markers of metabolic phenotype. J Clin Endocrinol Metab. 2009;94(3):827-32. 10.1210/jc.2008-1422 PMC2681283 19088165

[15] Yeap BB, Chubb SA, Flicker L, McCaul KA, Ebeling PR, Beilby JP, et al. Reduced serum total osteocalcin is associated with metabolic syndrome in older men via waist circumference, hyperglycemia, and triglyceride levels. Eur J Endocrinol. 2010;163(2):265-72. 10.1530/EJE-10-0414 20501596

[16] Kanazawa I, Yamaguchi T, Yamamoto M, Yamauchi M, Kurioka S, Yano S, et al. Serum osteocalcin level is associated with glucose metabolism and atherosclerosis parameters in type 2 diabetes mellitus. J Clin Endocrinol Metab. 2009;94(1):45-9. 10.1210/jc.2008-1455 18984661

[17] Goliasch G, Blessberger H, Azar D, Heinze G, Wojta J, Bieglmayer C, et al. Markers of bone metabolism in premature myocardial infarction (≤ 40 years of age). Bone. 2011;48(3):622-6. 10.1016/j.bone.2010.11.005 21078422

[18] Zhou M, Ma X, Li H, Pan X, Tang J, Gao Y, et al. Serum osteocalcin concentration in relation to glucose and lipid metabolism in Chinese individual. Eur J Endocrinol. 2009;161(5):723-9. 10.1530/EJE-09-0585 19671707

[19] García-Martín A, Cortés-Berdonces M, Luque-Fernández I, Rozas-Moreno P, Quesada-Charneco M, Muñoz-Torres M. Osteocalcin as a marker of metabolic risk in healthy postmenopausal women. Menopause. 2011;18(5):537-41. 10.1097/gme.0b013e3181f8565e 21178793

[20] Fernández-Real JM, Izquierdo M, Ortega F, Gorostiaga E, Gómez-Ambrosi J, Moreno-Navarrete JM, et al. The relationship of serum osteocalcin concentration to insulin secretion, sensitivity, and disposal with hypocaloric diet and resistance training. J Clin Endocrinol Metab. 2009;94(1):237-45. 10.1210/jc.2008-0270 18854399

[21] Saleem U, Mosley Jr TH, Kullo IJ. Serum osteocalcin is associated with measures of insulin resistance, adipokine levels, and the presence of metabolic syndrome. Arterioscler Thromb Vasc Biol. 2010;30(7):1474-8. 10.1161/ATVBAHA.110.204859 PMC2939910 20395593

[22] Bao Y, Ma X, Yang R, Wang F, Hao Y, Dou J, et al. Inverse relationship between serum osteocalcin levels and visceral fat area in Chinese men. J Clin Endocrinol Metab. 2013;98(1):345-51. 10.1210/jc.2012-2906 23162093

[23] Winhofer Y, Handisurya A, Tura A, Bittighofer C, Klein K, Schneider B, et al. Osteocalcin is related to enhanced insulin secretion in gestational diabetes mellitus. Diabetes Care. 2010;33(1): 139-43. 10.2337/dc09-1237 PMC2797959 19808925

[24] Hwang Y-C, Jeong I-K, Ahn KJ, Chung HY. The uncarboxylated form of osteocalcin is associated with improved glucose tolerance and enhanced beta-cell function in middle-aged male subjects. Diabetes Metab Res Rev. 2009;25(8):768-72. 10.1002/dmrr.1045 19877133

[25] Kanazawa I, Yamaguchi T, Yamauchi M, Yamamoto M, Kurioka S, Yano S, et al. Serum undercarboxylated osteocalcin was inversely associated with plasma glucose level and fat mass in type 2 diabetes mellitus. Osteoporos Int. 2011;22(1):187-94. 10.1007/s00198-010-1184-7 20165834

[26] Diaz-Lopez A, Bullo M, Juanola-Falgarona M, Martinez-Gonzalez MA, Estruch R, Covas M-I, et al. Reduced serum concentrations of carboxylated and undercarboxylated osteocalcin are associated with risk of developing type 2 diabetes mellitus in a high cardiovascular risk population: a nested case-control study. J Clin Endocrinol Metab. 2013;98(11):4524-31. 10.1210/jc.2013-2472 24037881

[27] Pollock NK, Bernard PJ, Gower BA, Gundberg CM, Wenger K, Misra S. Lower uncarboxylated osteocalcin concentrations in children with prediabetes is associated with beta-cell function. J Clin Endocrinol Metab. 2011;96(7):E1092-9. 10.1210/jc.2010-2731 PMC3135188 21508147

[28] Reinehr T, Roth CL. A new link between skeleton, obesity and insulin resistance: relationships between osteocalcin, leptin and insulin resistance in obese children before and after weight loss. Int J Obes (Lond). 2010;34(5):852-8. 10.1038/ijo.2009.282 20065970

[29] Yeap BB, Alfonso H, Chubb SA, Gauci R, Byrnes E, Beilby JP, et al. Higher serum undercarboxylated osteocalcin and other bone turnover markers are associated with reduced diabetes risk and lower estradiol concentrations in older men. J Clin Endocrinol Metab. 2015;100(1):63-71. 10.1210/jc.2014-3019 25365314

[30] Shea MK, Gundberg CM, Meigs JB, Dallal GE, Saltzman E, Yoshi-da M, Jacques PF, Booth SL. Gamma-carboxylation of osteocalcin and insulin resistance in older men and women. Am J Clin Nutr. 2009;90(5):1230-5. 10.3945/ajcn.2009.28151 PMC2762158 19776145

[31] Mori K, Emoto M, Motoyama K, Lee E, Yamada S, Morioka T, et al. Undercarboxylated osteocalcin does not correlate with insulin resistance as assessed by euglycemic hyperinsulinemic clamp technique in patients with type 2 diabetes mellitus. Diabetol Metab Syndr. 2012;4(1):53. 10.1186/1758-5996-4-53 PMC3565869 23249601

[32] Fodor D, Vesa S, Albu A, Simon S, Craciun A, Muntean L. The relationship between the metabolic syndrome and its components and bone status in postmenopausal women. Acta Physiol Hung. 2014;101(2):216-27. 10.1556/APhysiol.101.2014.2.10 24901081

[33] Schafer AL, Sellmeyer DE, Schwartz AV, Rosen CJ, Vittinghoff E, Palermo L, et al. Change in undercarboxylated osteocalcin is associated with changes in body weight, fat mass, and adiponectin: parathyroid hormone (1-84) or alendronate therapy in postmenopausal women with osteoporosis (the PaTH study). J Clin Endocrinol Metab. 2011;96(12):E1982-9. 10.1210/jc.2011-0587 PMC3232610 21994958

[34] Vergnaud P, Garnero P, Meunier PJ, Bréart G, Kamihagi K, Delmas PD. Undercarboxylated osteocalcin measured with a specific immunoassay predicts hip fracture in elderly women: the EPIDOS Study. J Clin Endocrinol Metab. 1997;82(3):719-24. 10.1210/jcem.82.3.3805 9062471

[35] Nimptsch K, Hailer S, Rohrmann S, Gedrich K, Wolfram G, Linseisen J. Determinants and correlates of serum undercarboxylated osteocalcin. Ann Nutr Metab. 2007;51(6):563-70. 10.1159/000114211 18227625

[36] Aonuma H, Miyakoshi N, Hongo M, Kasukawa Y, Shimada Y. Low serum levels of undercarboxylated osteocalcin in postmenopausal osteoporotic women receiving an inhibitor of bone resorption. Tohoku J Exp Med. 2009;218(3):201-5. 10.1620/tjem.218.201 19561390

[37] Alberti KG, Zimmet P, Shaw J; IDF Epidemiology Task Force Consensus Group. The metabolic syndrome - a new worldwide definition. Lancet. 2005;366(9491):1059-62. 10.1016/S0140-6736(05)67402-8 16182882

[38] Matthews DR, Hosker JP, Rudenski AS, Naylor BA, Treacher DF, Turner RC. Homeostasis model assessment: insulin resistance and beta-cell function from fasting plasma glucose and insulin concentrations in man. Diabetologia. 1985 Jul;28(7):412-9. 10.1007/BF00280883 3899825

[39] Paz-Filho GJ, Volaco A, Suplicy HL, Radominski RB, Boguszewski CL. Decrease in leptin production by the adipose tissue in obesity associated with severe metabolic syndrome. Arq Bras Endocrinol Metabol. 2009;53(9):1088-95. 10.1590/s0004-27302009000900005 20126866

[40] Miura M. Biochemical markers of bone turnover. New aspect. An automated assay for measuring bone turnover markers. Clin Calcium. 2009;19(8):1160-9. 19638700

[41] Szulc P, Chapuy MC, Meunier PJ, Delmas PD. Serum undercarboxylated osteocalcin is a marker of the risk of hip fracture in elderly women. J Clin Invest. 1993;91(4):1769-74. 10.1172/JCI116387 PMC288157 8473517

[42] Levinger I, Zebaze R, Jerums G, Hare DL, Selig S, Seeman E. The effect of acute exercise on undercarboxylated osteocalcin in obese men. Osteoporos Int. 2011;22(5):1621-6. 10.1007/s00198-010-1370-7 20734028

[43] Pollock NK, Bernard PJ, Wenger K, Misra S, Gower BA, Allison JD, et al. Lower bone mass in prepubertal overweight children with prediabetes. J Bone Miner Res. 2010;25(12):2484-93. 10.1002/jbmr.184 PMC3122138 20641032

